# Emergencies—Euthanasia

**Published:** 1887-04

**Authors:** J. T. Kent

**Affiliations:** St. Louis


### EMERGENCIES—EUTHANASIA.

Professor J. T. Kent, M. D., St. Louis.

I am frequently asked, what should be done in times of great suffering for immediate relief? To those who desire to obtain reliable information, and who wish to practice in accordance with our principles, I would say, take the symptoms of each individual case and select the remedy capable of producing similar symptoms. In a general way this is all that would be expected of me for an answer to the question, by those who are conversant with materia medica.

Consumptives often suffer greatly when left to themselves, and some medical practitioners, knowing no better way, give Morphine and other stupefying agents, thinking that they allay human suffering.

This kind of practice cannot be too strongly condemned. Firstly, it is an acknowledgment that our law is not all-embracing; secondly, it is the *poorest kind of relief to the patient.* But I would not deprive medical practitioners of all means of relief for their patients, without furnishing as good or better ones.

The consumptive, when going down the last grade, needs the comfort of a true *healing art,* and not the makeshifts of mongrelism or allopathy. The homœopathic remedy is all that he, who knows how to use it, needs to allay the severest distress. Every true homœopathist knows the value of these wonderful remedies.

A few hints may not be out of place.

When the hectic fever, that so rapidly burns the patient up, is in full blast; the hot afternoon skin, the night sweat, the constant burning thirst, the red spot on the cheek, the diarrhœa, the stool escapes when coughing, *the intense fever* P. M.; the constriction of the chest, suffocation; then should *Phos.*, *very high*, be administered, but *never repeated.* An aggravation will follow, but it must not be meddled with, as it will soon pass off, leaving the patient free from fever, and he will go on till death, many times, comfortably. *It is the regrettable meddling that causes the dying man so much misery.*

The distressed suffocation and inward distress in chest and stomach, streaming perspiration, great sinking; must have the clothing away from neck, chest, abdomen, ghastly countenance, and choking, call for *Lachesis*, and it may be given as often as occasion requires, but to give satisfaction and prompt relief, not lower than two hundredth.

To this ghastly picture, if we add, he is covered with a cold sweat, and there is one on either side of the bed fanning him, and the abdomen is distended with flatus, and the breath is cold, *Carbo v.* in water every hour for six hours, and stopped, will give rest and beatitude with many thanks.

But the time is yet coming when even these remedies will not serve us.

The ghastliness of the picture has not been changed, and to it we have added the pains of dying cells—death pains, the last suffering. Such pains come on when mortification begins. If it is in the abdomen, we may avert it by differentiating between *Arsenicum* and *Secale*, but if this pain comes in the last stage of consumptive changes, we are beyond these remedies. Much later there is a remedy, and it is *Tarentula cubensis.* It soothes the dying sufferer as I have never seen any other remedy do.

I have seen *Ars.*, *Carbo v.*, *Lyc.*, *Lach.*, act kindly and quiet the last horrors, but *Tarentula cubensis* goes beyond these. I have lately administered it in the thirtieth cent. potency.

When death is inevitable, the first named remedies seem to be mostly indicated, but no longer act, and the friends say, "Doctor, can't you do something to relieve that horrible suffering?" the pain, the rattling in the chest, with no power to throw the mucus out; the patient has but a few hours to suffer, but can be made as quiet as with the terrible Morphine in a very few minutes by the *Tarentula* thirtieth.

I believe that no physician would use a narcotic if he only knew a better way.

What is more inhuman than to leave the suffering patient in his last moments to writhe in the agonies of dissolution, surrounded by weeping friends? The true physician will embrace the opportunity to exercise his skill at these moments. It has come to pass that I am invited frequently to stand at the bed of *moribund* patients, whom I never attended during their curable ills, and as many times do I thank the Grand Master for the wonderful means of allaying the pangs of the flesh, without resort to the necessity of departing from that law which I have so many times pronounced universal; even in the last moments—a euthanasia.—*St. Louis Periscope.*

